# The Evolving Role of Immunotherapy in Stage III Non-Small Cell Lung Cancer

**DOI:** 10.3390/curroncol28060451

**Published:** 2021-12-16

**Authors:** Kirstin Perdrizet, Parneet K. Cheema

**Affiliations:** 1 Division of Medical Oncology, University of Toronto, Toronto, ON M5S 1A8, Canada; Parneet.Cheema@williamoslerhs.ca; 2 Medical Oncology/Hematology, William Osler Health System, Brampton, ON L6R 3J7, Canada

**Keywords:** immunotherapy, NSCLC, Stage III, adjuvant, neoadjuvant, concurrent chemotherapy and radiation, checkpoint inhibitors

## Abstract

The management of Stage III non-small cell lung cancer (NSCLC) is complex and requires multidisciplinary input. Since the publication of the PACIFIC trial (consolidative durvalumab post concurrent chemotherapy and radiation in Stage III disease) which showed improved survival for patients in the immunotherapy arm, there has been much interest in the use of immunotherapy in the Stage III setting. In this review, we explore the biologic and clinical rationale for the use of immunotherapy in Stage III NSCLC, present previously published and upcoming data in the neoadjuvant, adjuvant, and concurrent realms of Stage III management, and discuss unanswered questions and challenges moving forward.

## 1. Introduction

Approximately 20–30% of patients diagnosed with non-small cell lung cancer (NSCLC) present with Stage III disease [1]. The management of Stage III NSCLC is complex and can include surgical resection, chemotherapy, radiation as well as a combination of all modalities. This is because Stage III NSCLC encompasses a heterogeneous group of tumour presentations defined as having spread locoregionally through primary tumour extension into extrapulmonary structures (T3/4) and involving hilar or mediastinal lymph nodes (N1–3), but having no evidence of distant metastases (M0) [2].

Surgical resection can offer a curative option for Stage IIIA disease, although 5-year overall survival (OS) for resected Stage IIIA patients is only 36% leaving significant room for improvement [2]. Prior to the IMpower010 trial and the use of immunotherapy in the adjuvant setting, the treatment standard for patients with resected Stage III NSCLC was adjuvant platinum-based doublet chemotherapy. The lung adjuvant cisplatin evaluation (LACE) meta-analysis of five randomised Phase 3 trials reported that adjuvant chemotherapy could improve 5-year OS by only 5.4% in patients after complete resection of NSCLC (HR = 0.83 for Stage III patients) [3]. Similarly, when chemotherapy is given in the neoadjuvant setting the absolute improvement of OS is also approximately 5% at 5 years [4].

Although patients with Stage III NSCLC have disease that is confined to the thorax, surgery as the primary modality for treatment is rarely feasible (other than for select patients for example, T3N1M0 and T4N0M0), and has not been shown to be superior to concurrent chemotherapy and radiation (cCRT), which has remained the standard of care curative intent strategy for unresectable Stage III NSCLC [5].

As with the treatment of many malignancies, advances in the management of early disease are often extrapolated from successes in the treatment of later stages. Immune check point inhibitors (ICIs) have been particularly exciting, as many patients have durable treatment effect, commonly referred to as the “tail of the curve”, highlighting long term survivors. Given this, introducing ICIs earlier in the disease state was undertaken. The role of immunotherapy (IO) with ICIs to the management of Stage III disease was solidified when the PACIFIC trial demonstrated that the PD-L1 inhibitor durvalumab given as consolidation therapy following cCRT substantially improved progression free survival (PFS) and OS, with 5-year survival reported as 43% [6]. More recently, adjuvant data from the IMpower010 revealed the PD-L1 inhibitor atezolizumab improved disease-free survival (DFS) following resection of PD-L1 positive Stage II and III NSCLC, and has supported expanding role of ICIs in the adjuvant setting [7]. Given these recent advances and little change in the adjuvant treatment landscape for NSCLC since the widespread use of adjuvant platinum doublet chemotherapy, trials of ICIs in the surgical (neoadjuvant and adjuvant) and non-surgical (concurrently or following completion of cCRT) have been initiated.

The purpose of this review is to explore the current role of IO with ICIs in the Stage III NSCLC setting, review treatment implications for patients, and discuss challenges and unanswered questions moving forward.

## 2. Surgically Resectable Stage III NSCLC

### 2.1. Adjuvant

Adjuvant chemotherapy has been the only treatment shown to improve overall survival for patients with resected NSCLC, although benefits are modest. As a contemporary benchmark to quantify newly discovered improvements, the addition of postoperative adjuvant chemotherapy for early-stage NSCLC improved cure rates by 5%, which led to its widely accepted use [3]. There is also evidence that platinum and vinorelbine based adjuvant therapy could improve outcomes by 10–15% for patients with Stage II and III disease [8].

The post-surgical tumour microenvironment is immunosuppressive and postulated to be one of the reasons metastases can take hold. Surgical intervention alters cytokine levels, stress hormones, growth factor release, and the production of clotting factors [9]. This can lead to increased infiltration of regulatory immune cells, heightened expression of PD-1, decreased T-cell proliferation, and impaired NK-cell cytotoxicity, resulting in an immunosuppressive environment [9]. Therefore, mechanisms that can increase the immune response, such as ICIs, as well as the gains in OS realized with ICIs in the metastatic setting, have led to multiple trials in the adjuvant setting investigating whether improved cure rates can be achieved.

#### 2.1.1. Adjuvant ICIs

IMpower010 is the first Phase 3 trial of adjuvant ICIs to be presented, and there are currently multiple Phase 3 randomized trials with adjuvant ICIs underway (Table 1) [7,10]. IMpower010 randomized patients with Stage IB (>4 cm)-IIIA NSCLC to adjuvant atezolizumab or best supportive care after adjuvant platinum-based chemotherapy. Receipt of adjuvant chemotherapy was required for inclusion in the study. Patients were not excluded with driver mutations, and 41% of patients had Stage IIIA disease. The primary end point was DFS which was tested hierarchically (Stage II-IIIA patients with PD-L1 > 1%, all randomized Stage II-IIIA patients, ITT population Stage IB-IIIIA). Secondary endpoints included OS, DFS in PD-L1 > 50% Stage II-IIIA, and 3y/5y DFS is all three populations. In the PD-L1 positive Stage II-IIIA population the median DFS was not reached (NR) (36.1, NR), vs. 35.3 months (29, NR) with a HR of 0.66 (95% CI 0.50–0.88, *p* = 0.004). In all randomized Stage II-IIIA patients the DFS was 42.3 (36.0, NR) in the atezolizumab group vs. 35.3 (30.4, 46.4) in the best supportive care group with a HR of 0.79 (95% CI 0.64–0.96). In the ITT population the threshold for statistical significance was not met at the interim analysis. OS was not formally tested. There were no new safety signals.

Given the DFS benefit for patients with surgically resected PD-L1 positive Stage II-IIIA NSCLC, many feel that the IMpower010 results are practice changing and have defined a new standard of care. The United States Food and Drug Administration (US-FDA) has recently approved atezolizumab for the adjuvant treatment of surgically resected PD-L1 positive Stage II or III NSCLC. There is, however, yet to be regulatory approval for this indication in other jurisdictions.

#### 2.1.2. Challenges and Unanswered Questions: Adjuvant ICIs

As data continues to mature, it will be paramount to identify which patient subgroups benefit from adjuvant ICIs. In the IMpower010 trial patients with Stage II-IIIA PD-L1 positive disease had an improved HR compared with all Stage II-IIIA patients, however the data for the PD-L1 negative subgroup was not presented. In the subgroup analysis, almost all subgroups seemed to benefit from adjuvant atezolizumab except for those that received carboplatin/gemcitabine, those that received a bilobectomy, and those with *ALK* fusions [7]. Interestingly, patients with *EGFR* mutations also seemed to derive benefit, although numbers in the *EGFR* and *ALK* subgroups were very small. Ongoing Phase III trials and subsequent subgroup analyses will be paramount in determining which patients should be offered adjuvant ICIs (Table 1). The IMpower010 data must also be interpreted in the context of recent regulatory approvals for adjuvant targeted therapy in *EGFR* positive NSCLC. The ADAURA trial of adjuvant osimertinib in surgically resected Stage IB-IIIA NSCLC revealed a substantial DFS benefit in those with Stage II or IIIA disease; at 24 months, 90% of the patients in the osimertinib group (95% CI 84–93) and 44% of those in the placebo group (95% CI 37–51) were alive and disease-free (HR 0.17; 99% CI 0.11–0.26; *p* < 0.001) [11]. Although *EGFR* positive patients were represented in the IMpower010 trial and in the forest plot favored the atezolizumab arm, numbers were small (9% of patients in the PD-L1 positive Stage II-III group, although 50% of patients had unknown *EGFR* status). Many clinicians have moved away from using ICIs in this population (in Stage IV disease or as consolidation post cCRT) given the minimal benefit observed in multiple pooled and individual analyses [12,13]. Although *EGFR* positive patients were included in IMpower010, given the results of the ADAURA study and questionable benefit of ICIs in later line treatment of *EGFR* positive NSCLC, it is likely that most clinicians will opt for adjuvant osimertinib in these patients.

Lastly, there are other groups of patients with Stage IIIA NSCLC where the role of adjuvant ICIs remain unknown. Trimodality therapy (neoadjuvant cCRT followed by surgical resection) is a frequently used treatment in Stage IIIA disease. At the present time questions remain unanswered regarding the role of adjuvant immunotherapy following trimodality therapy, as these patients are not included in the ongoing Phase 3 adjuvant trials (Table 1).

### 2.2. Neoadjuvant

Neoadjuvant therapy has advantages such as reducing tumour size, increasing operability, and early eradication of micrometastases. It also offers the advantage of prompt initiation of treatment in regions that have lengthy surgical wait times. The rationale for IO prior to surgery is to leverage the primary tumour as an antigen source for expansion and activation of tumor-specific T-cells, resulting in destruction of micrometastases. However, this approach runs the risk of delaying definitive surgical management, either due to disease progression if treatment is ineffective, or if toxicity related to IO precludes it.

A review and meta-analysis conducted by the NSCLC Meta-analysis Collaborative Group, showed that neoadjuvant chemotherapy followed by surgery for Stage I to III NSCLC improved the 5-year OS by 5% (HR 0.87, 95% CI 0.78–0.96, *p* = 0.007) compared to surgery alone. Those with Stage III NSCLC had an absolute OS improvement from 20% to 25% [4]. The type of chemotherapy or scheduling did not appear to influence outcomes and neoadjuvant chemotherapy appeared safe with no excess of early mortality as a result of deferred surgery.

An endpoint that has been used in neoadjuvant trials has been pathologic response. Major pathologic response (MPR) is defined as less than or equal to 10% remaining viable tumour cells (RVTs) in the surgical resection specimen, and a pathologic complete response (pCR) is defined as no RVTs in the surgical resection specimen. MPR and pCR correlate with OS in the neoadjuvant chemotherapy setting [14,15,16]. MPR and pCR rates are low with neoadjuvant chemotherapy and are 20% and 4% respectively [15,17].

#### 2.2.1. Neoadjuvant ICI Monotherapy

The landmark study by Forde et al. set the stage for the use of ICIs in the neoadjuvant setting (Table 2) [18]. The authors reported on 21 patients with Stage I-III NSCLC (7 which were Stage IIIA) treated with 2 doses of nivolumab (3 mg/kg q2 weekly) followed by surgical resection 4 weeks after start of nivolumab. The primary endpoints were safety and feasibility, which were met. All grade treatment related adverse events (TRAEs) occurred in 23% and there were no treatment related surgical delays. The most impressive finding was that 9 of the 20 patients (45%) (1 excluded due to small cell histology) that had tumour resection had an MPR, of which 3 were a pCR (15%). Responses were seen irrespective of PD-L1 status [18].

A second neoadjuvant trial with the PD-1 inhibitor sintilimab found similar results [19]. Two doses of sintilimab were administered in the neoadjuvant setting (45% Stage IIIA/B (N2)). Thirty-seven patients were amenable to surgical resection of which 41% had an MPR and 16% a pCR. TRAEs of Grade 3 or higher occurred in 4 patients (10%). Although responses were seen irrespective of PD-L1 status, unique to this trial was that MPR was found to be correlated with PD-L1 status of stromal cells (as opposed to tumour cells) (*p* = 0.047). Interestingly all patients that had an MPR were of squamous cell histology, although most patients enrolled were of SCC histology (80%) [19].

Subsequent trials of neoadjuvant monotherapy PD-(L)1 inhibitors in resectable NSCLC patients were not as encouraging and found to have lower MPR rates of approximately 20%, similar to that seen with chemotherapy (Table 2) [20,21,22,23,24,25].

The largest trial with a single agent PD-L1 inhibitor was the LCMC3 trial [21]. This study evaluated 2 cycles of neoadjuvant PD-L1 inhibitor atezolizumab, followed by surgery and then 1 year of adjuvant atezolizumab. Of the 181 patients enrolled, 85 (47%) were Stage IIIA/B. The primary endpoint of the study was MPR in the *EGFR*/*ALK* negative population, and was met (20% MPR, and 7% CR). Concerning however, was that at the time of surgery 16% of patients were deemed surgically unresectable (12% preoperatively and 4% intraoperatively). Grade 3–4 TRAEs occurred in 5% of patients. Responses were seen across all PD-L1 subsets but more likely to occur in those with high PD-L1 expression (using the 22C3 clone) (MPR 35% in PD-L1 > 50%, and 11% PD-L1 < 50%, *p* = 0.04) [21].

The IONESCO study of neoadjuvant durvalumab is the only study of ICIs in the neoadjuvant setting that was halted early due to safety concerns given increased 90-day postoperative mortality [22]. The study included patients with NSCLC and Stage IB (>4 cm)-IIIA (non N2) disease (1 patient had Stage III disease) treated with 3 cycles of Q2 weekly durvalumab prior to surgery. The primary endpoint was R0 resection with secondary endpoints of safety, OS, DFS, MPR, time from first durvalumab to surgery, and response rate (RR). Forty-six patients were eligible for the study and 43 patients underwent surgical resection (1 patient progressed, 1 had pleural invasion, 1 had esophageal invasion). The study was halted due to an excess of 90-day mortality after 4 patients died; no patients died of immune related adverse events (irAEs) and deaths were primarily attributed to pre-existing comorbidities. No grade >3 durvalumab-related adverse events were observed. Analysis of the recruited cohort revealed a 90% R0 resection rate, and MPR in 19% of patients. This trial also demonstrated that MPR was significantly associated with DFS (*p* = 0.02), and that the extent of the pathological response (measured as % of RVT) is an independent prognostic factor for OS and DFS [26]. In a multivariable analysis the risk of death increased by 64% for each RVT unit (unit defined as 10% increase in RVT), demonstrating that RVT is a continuous variable [26].

#### 2.2.2. Neoadjuvant Combination ICIs

The NEOSTAR study was the first trial to evaluate ICI combinations in the neoadjuvant setting [20]. Forty-four patients with resectable Stage I-IIIA (20% with Stage III) received 2 doses of nivolumab (*n* = 23) or nivolumab in combination with ipilimumab the cytotoxic T-lymphocyte antigen (CTLA-4) inhibitor (*n* = 21). The primary endpoint was MPR and was higher in those that received combination ICIs (38%) compared with nivolumab monotherapy (22%). Responses in both arms were irrespective of PD-L1 status. Grade 3–5 TRAEs were low; 13% and 10% in those treated with nivolumab and nivolumab/ipilimumab respectively. Of the 44 patients, 5 did not go for resection. One in the nivolumab arm secondary to a TRAE, and 4 in the nivolumab/ipilimumab arm [progressive disease (*n* = 1), no longer eligible for surgery due to involvement of the carina (*n* = 1), non-treatment related causes (*n* = 2) [20].

#### 2.2.3. Neoadjuvant ICIs and Stereotactic Body Radiotherapy (SBRT)

SBRT might be a potent immunomodulatory in advanced NSCLC [27]. Given this, a Phase 2 trial comparing neoadjuvant durvalumab for 2 doses vs. neoadjuvant durvalumab +SBRT (8gy × 3 consecutive fractions to the primary tumour), with a primary end point of MPR was conducted [28]. Sixty patients with Stage I-III disease were enrolled in the study. Surgical resection was performed in 86% of patients (26/group). MPR occurred in 7% of patients treated with durvalumab monotherapy and 53% in the SBRT +durvalumab group [odds ratio for MPR with durvalumab/SBRT vs. durvalumab monotherapy was 16.0 (95% CI 3.2–79.6, *p* < 0.0001)]. Grade 3/4 AEs occurred in 18% of all patients (5 in the durvalumab arm and 6 in the durvalumab/SBRT group).

#### 2.2.4. Neoadjuvant ICIs with Chemotherapy

ICIs and chemotherapy have shown to have a synergistic effect in advanced NSCLC and the combination has proven benefit across PD-L1 expression levels compared to chemotherapy alone [29,30]. Patients receiving ICIs and chemotherapy also seem to have improved PFS with subsequent therapies, indicating there could be a lingering benefit from the upfront ICI chemotherapy combination [31,32].

The Phase 2 NADIM trial was the first neoadjuvant study to report outcomes of ICIs with chemotherapy [33]. This study included 46 patients with resectable Stage IIIA NSCLC who underwent neoadjuvant treatment with carboplatin/paclitaxel/nivolumab (q3 weekly × 3 cycles) before surgical resection, followed by adjuvant nivolumab for 1 year. The primary endpoint was PFS at 24 months. Forty-one patients underwent surgical resection (2 declined surgery, 3 were deemed surgically unresectable). PFS at 24 months was 77% and OS was 90%. TRAEs grade >3 occurred in 30% of patients but did not lead to death or surgical delay. This approach led to high MPR rates of 83% and high pCR rates of 63% in those that went to resection. MPR and pCR were associated with increased PFS and OS. Patients with an MPR or pCR had PFS at 24 months of 88% compared to 57% in those with an incomplete pathologic response (*p* = 0.010) [33].

Two other small neoadjuvant trials of ICIs with chemotherapy have reported similar MPR rates. This included neoadjuvant atezolizumab with platinum-based chemotherapy × 4 cycles (*n* = 30) resulting in MPR rates of 57%, and neoadjuvant PD-1 inhibitor toripalimab in combination with platinum-based chemotherapy for 3 cycles in patients with Stage IIIA/B NSCLC yielding a MPR rate of 67% [34,35]. Sequential ICI and chemotherapy was further studied in the SAKK 16/14 study, a single arm Phase 2 trial of neoadjuvant cisplatin/docetaxel for 3 cycles followed by sequential durvalumab (q2 weekly × 2 doses) [36,37]. This approach also led to encouraging outcomes in the 68 patients enrolled (67 included in the analysis), of which 62% achieved a MPR and 18% a pCR.

Although the results with various approaches to neoadjuvant IO have been promising regarding achieving a MPR, the results of ongoing Phase 3 trials are required until this is considered a standard of care option (Table 3) and currently neoadjuvant ICIs should only be used in a clinical trial setting. Checkmate 816 was the first randomized Phase 3 trial of neoadjuvant ICI with chemotherapy to report early outcomes [38]. This trial randomized 358 patients to 3 cycles of neoadjuvant platinum doublet versus the combination of nivolumab with platinum doublet in Stage IB-IIIA (64% Stage IIIA) NSCLC, in which half were PD-L1 ≥1%. The primary endpoints were pCR and event free survival (EFS), of which only pCR has been reported in the literature. Nivolumab/platinum doublet yielded pCR rates of 24% versus 2.2% for chemotherapy alone. Definitive surgery was performed in 83% in the nivolumab/platinum doublet arm compared to 75% in those that received chemotherapy alone. Surgery was canceled due to adverse events in 1% per arm, and 7% due to progressive disease/8% miscellaneous reasons in the nivolumab/platinum doublet arm and 9% progressive disease/11% miscellaneous reasons in the chemotherapy arm. Median RVTs were substantially lower in the nivolumab/platinum doublet arm of 10% compared to 74% in the platinum doublet arm. The complexity of surgery did not increase with nivolumab as the median duration of the surgery was similar between both groups. There was no difference in 90-day surgical related complications (Grade 3–4 11% with nivolumab/platinum doublet and 15% with chemotherapy alone).

#### 2.2.5. Challenges and Unanswered Questions: Neoadjuvant ICIs

One of the challenges that has arisen from using neoadjuvant ICIs is assessing response to treatment. Despite an MPR of 45% in the Forde study, there were only two patients with a radiologic partial response and two patients that had increased radiographic tumor size were found to have minimal or no residual tumour in the surgical specimen [18]. This was due to immune-cell infiltration into the tumour, rather than tumour growth [39]. In an ad hoc analysis of the NEOSTAR trial, a “nodal immune flare” (NIF) phenomenon was observed in which patients treated with neoadjuvant ICIs demonstrate radiologically abnormal nodes post-therapy that upon pathological evaluation were devoid of cancer and demonstrated de novo non-caseating granulomas [40]. This occurred in approximately 16% of patients treated with ICIs. There is therefore a need to distinguish NIF from true nodal progression within the mediastinum with invasive pathologic evaluation.

There are also multiple unanswered questions in this space. PD-L1 has been relied upon as a biomarker in the advanced setting but has not been shown to be as reliable or predictive of response in the neoadjuvant setting. In the Forde study, there was significant correlation between pre-treatment tumour mutational burden (TMB) and pathological response [18]. Primary endpoints used in most of the aforementioned neoadjuvant studies have been MRP and pCR, and although correlate with OS they remain surrogate endpoints for which we do not have long term robust data to rely on. The data generated to support the use of MPR and pCR were generated in the age of chemotherapy, and have not been validated in the setting of targeted therapy or immunotherapy. Lastly, we have not identified the optimal number/duration of doses prior to surgical resection, which will be important to determine to minimize risk of toxicity or progression leading to inoperability. Given the above the use of neoadjuvant ICIs should remain limited to the clinical trial setting until more data are available.

## 3. Surgically Unresectable Stage III NSCLC

### 3.1. ICIs with cCRT

There is clinical and preclinical evidence to suggest the use of radiation therapy in combination with ICIs may enhance antigen exposure and ICI response in solid tumours.

Although ICIs have changed the treatment landscape of NSCLC, there remain tumours that do not respond to ICIs, the cause of which is postulated through multiple resistance mechanisms such as low neoantigen burden, reliance on anerobic metabolism, and down regulation of MHC expression, among others [41]. Radiation has been shown to alter the tumour microenvironment, including alterations in antigen presentation and upregulation of PD-L1 expression [42]. Radiation has also been demonstrated to increase tumour infiltrating lymphocytes (TILs) in preclinical models [43]. There is also retrospective clinical data to suggest that induction or concurrent ICI therapy with RT can improve OS in patients when given concurrently or within 30 days across multiple tumour subtypes [27]. Due to this pre-clinical and clinical data, the use of ICIs concurrently or sequentially with cCRT is under substantial investigation in the setting of unresectable Stage III NSCLC.

Prior to the publication of the PACIFIC trial, 5-year overall survival for patients Stage III NSCLC treated with curative intent cCRT was 20–30% [44]. The addition of ICI post chemotherapy with durvalumab in the PACIFIC trial increased the 5-year OS to 43% [6]. PACIFIC randomized 713 patients (2:1) to receive durvalumab or placebo respectively after cCRT. The primary endpoints were OS and PFS in the ITT population [45]. With a median follow up of 34 months in all patients, the median OS was 47.5 vs. 29.1 months in the durvalumab and placebo groups respectively (HR 0.72, 95% CI 0.59–0.89) and median PFS 16.9 vs. 5.6 months (HR 0.55, 95% CI 0.45–0.68). When patients were stratified by PD-L1 status (unplanned non-prespecified analysis) patients with PD-L1 negative disease had an elevated hazard ratio for death (HR 1.38, 95% CI 0.79–2.34). Despite this being an unplanned subgroup analysis, consolidative durvalumab is not uniformly approved by all regulatory authorities for the PD-L1 negative population. The main safety concern with the PACIFIC regimen was the rate of pneumonitis, as this can occur with ICIs and with radiation. Overall, the treatment was well tolerated with a minor elevated risk of pneumonitis in the durvalumab group (any grade 33.9% vs. 24.8%), but the incidence of more serious (grade 3+) pneumonitis occurred in similar rates in both arms (3.4% vs. 2.6% of the durvalumab and placebo groups respectively).

Nivolumab and Pembrolizumab have both been studied in the consolidative (post cCRT) setting for Stage III NSCLC [46,47]. A study of consolidative nivolumab vs. placebo after cCRT was halted early after the publication of PACIFIC due to the control arm being obsolete [46]. A single arm Phase 2 study of pembrolizumab for 1 year post cCRT for Stage IIIA/B NSCLC has also been reported [47]. The primary endpoints of the study were time to death or distant metastatic disease (TMDD), with PFS/OS/safety as secondary endpoints. The trial enrolled 93 patients with a median follow up of 32.2 months, the median TMDD was 30.7 months (95% CI 18.7-NR), and this was significantly longer compared to historical controls (*p* < 0.0001). The median PFS was 18.7 months (95% CI, 12.4–33.8), and the median OS was 35.8 months (95% CI, 24.2-NR). Unlike the PACIFIC trial however, when stratified by PD-L1 status (PD-L1 > 1% vs. negative) there was no difference seen in TMDD, PFS or OS. The treatment was well tolerated, and Grade 2 or higher pneumonitis was noted in 17.2% of participants.

Currently, only Phase 1 and 2 trials have early safety and efficacy data to report for combination ICIs and cCRT, and there are currently many ongoing Phase 3 trials of ICIs with cCRT for NSCLC (Table 4). The NICOLAS trial is a Phase 2 study primarily evaluating the safety of nivolumab added to cCRT for Stage III NSCLC, with a hierarchical testing model first to evaluate safety (6-month post RT rate of grade greater than or equal to 3 pneumonitis), and if met, then efficacy (PFS and OS) [48]. An interim safety analysis was planned when the first 21 patients had 3 months of follow-up post RT, and an early positive safety conclusion was reached. For the efficacy analysis a total of 79 patients were enrolled and the median PFS was 12.7 months (95% CI 10.1–22.8) and the 1-year PFS rate was 53.7% (95% CI 42.0%–64.0%) with a median follow-up of 21.0 months (IQR 15.8–25.8). However, due to the hierarchical testing model, the pre-specified 1-year PFS rate of 45% was not rejected (*p* = 0.23). The median OS was 38.8 months (95% CI 26.8–NR) and a 2-year OS rate was 63.7% (95% CI 51.9%–73.4%).

A study of multiple combinations of pembrolizumab with cCRT was conducted in the Phase 1 setting, primarily to evaluate dose limiting pneumonitis (at least Grade 4) [49]. Pembrolizumab was combined with cCRT with varied timing (prior to, during and after) and doses (200 mg and 100 mg). Twenty-one patients enrolled in the various cohorts were included in the analysis, and there were no dose limiting toxicities. In the safety expansion cohort of pembrolizumab 200 mg give day 1 of cCRT, there was one case of Grade 5 pneumonitis. Grade 3 or higher irAEs occurred in 18%. Median PFS for patients who received at least 1 dose of pembrolizumab (*n* = 21) was 18.7 months (95% CI 11.8–29.4 mos). A single arm Phase 2 study (KEYNOTE-799) of pembrolizumab with cCRT followed by consolidative pembrolizumab also revealed the safety and efficacy of this combination [50]. Patients received one cycle of induction carboplatin, paclitaxel and pembrolizumab, followed by concurrent weekly paclitaxel and carboplatin for 6 weeks (pembrolizumab given every 3 weeks for 2 doses), then consolidative pembrolizumab for 14 doses. ORR and incidence of Grade 3 or higher pneumonitis were the primary endpoints. The study enrolled 216 patients in two cohorts, Cohort A non-squamous and squamous histologies, and Cohort B squamous histology (112 in Cohort A and 104 in Cohort B). ORR) was 70.5% (95% CI, 61.2–78.8%) in Cohort A and 70.6% (95% CI, 60.7–79.2%) in Cohort B. Grade ≥3 pneumonitis occurred in 9 patients (8.0%) in Cohort A and 7 patients (6.9%) in Cohort B.

Atezolizumab has also been studied in a single arm Phase 2 (*n* = 30) of concurrent atezolizumab (2 cycles) with cCRT, followed by maintenance atezolizumab for 1 year [51]. The primary endpoint was safety and tolerability, and secondary end points of PFS and OS. Grade 2 or higher pneumonitis occurred in 16%. The median PFS was 13.2 months, and the median OS was not reached at a median follow up time of 15.1 months. PD-L1 expression was not found to be related to PFS.

### 3.2. ICIs Post Sequential Chemotherapy and Radiation (sCRT)

There is also benefit of ICIs in patients that received sCRT. The recently presented study GEMSTONE-301 is a Phase 3 trial of sugemalimab (anti-PD-L1) as consolidation for 1 year after chemotherapy and radiation (either cCRT or sCRT) compared with placebo [52]. Approximately 33% of patients received sequential therapy. The 18-month PFS was 39% vs. 23%, in the sugemalimab and placebo arms respectively. Consistent PFS benefit was observed regardless of whether patients received sCRT (median PFS 8.1 vs. 4.1 months, HR 0.59) or cCRT (median PFS 10.5 vs. 6.4 months, HR 0.66).

Patients receiving sCRT were also included in the PACIFIC-R study (data from the real world post PACIFIC study durvalumab access program), and made up 14.3% of the study population [53]. The median PFS was 19.4 months (95% CI 12.4–25.3) with sCRT, which when compared to historical controls is substantially improved (median PFS was 5.6 months in the control arm of the PACIFIC trial).

### 3.3. Challenges and Unanswered Questions: ICIs with cCRT

Based on the aforementioned trials, there are no major safety signals that have been identified with ICIs delivered concurrently with cCRT in unresectable NSCLC. Rates of pneumonitis have been higher than with cCRT alone, however this seems to be driven by higher rates of low-grade pneumonitis, which is less clinically concerning. There is a recently published meta-analysis exploring this issue which suggests that concurrent ICI therapy with cCRT had greater rates of Grade 2 pneumonitis compared with sequential administration (23.0%, 95% CI 15.8–32.3% vs. 11.0%, 95% CI 6.6–17.8%, OR 0.42, *p* = 0.02) [54]. Whether the addition of ICIs concurrently with cCRT as opposed to post cCRT improves outcomes such as PFS or OS is yet to be determined. The ECOG-ACRIN EA5181 study will help clarify this question as it is a randomized Phase 3 trial of cCRT with or without durvalumab followed by 1 year of consolidative durvalumab [55]. This study, and other ongoing Phase 3 trials (Table 4) will help delineate whether ICIs should be given with cCRT or remain in the consolidative setting.

## 4. Conclusions: ICIs in the Management Stage III NSCLC

At the present time, durvalumab as consolidation therapy post cCRT is the only regulatory approved use of ICI therapy in the setting of unresectable Stage III NSCLC, and atezolizumab is the only approved adjuvant ICI therapy post-surgical resection of Stage III disease. The use of ICIs for Stage III disease, however, continues to evolve rapidly and has expanded to the neoadjuvant, concurrent and adjuvant space. Many of the unanswered questions surrounding the timing (with cCRT or post), benefits of different types/combinations of ICIs, and biomarkers predictive of response will begin to be answered as the currently ongoing Phase 3 trials read out (Table 1, Table 3 and Table 4). Many unanswered questions will remain however, such as whether neoadjuvant or adjuvant therapy should be favored, what the optimal duration of treatment is, which ICI(s) will be of most benefit, and whether there is a role for ICIs in the trimodality treatment setting. These questions will remain and need further study until definitive conclusions can be made.

## Figures and Tables

**Table 1:** Phase 3 clinical trials of adjuvant ICIs in surgically resected Stage III NSCLC.

Clinical Trial	*n*	Patient Population (AJCC 7th Unless Stated Otherwise)	Adjuvant Chemotherapy Required?	Study Arms	Endpoints
IMpower010 (NCT02486718)	1280	Stage IB ≥ 4 cm/II-IIIA	Yes	Atezolizumab Q3W × 1 year vs. best supportive care	Primary: DFS Secondary: OS, AEs
PEARLS/ KEYNOTE-091 (NCT02504372)	1177	Stage IB 4 cm/II-IIIA	Optional	Pembrolizumab Q3W × 1 year vs. placebo	Primary: DFS, Secondary OS, LCSS
ANVIL (NCT02595944)	903	Stage IB ≥ 4 cm/II-IIIA, *EGFR/ALK* excluded	Optional	Nivolumab Q4W × 1 year vs. Observation	Primary: DFS, OS Secondary: Toxicity
BR31 (NCT02273375)	1360	Stage IB ≥ 4 cm/II/IIIA	Optional. Neoadjuvant excluded	Durvalumab vs. placebo × 1 year	Primary: DFS TC ≥ 25% without *EGFR/ALK* mutations Secondary: DFS by PD-L1 and *EGFR/ALK*, OS, LCSS, Safety, Cost effectiveness
MeRmaidD-2 (NCT04642469)	284	Stage II-IIIA (AJCC. 8th) MRD+, *EGFR/ALK* excluded	Optional, allowed neoadjuvant	Durvalumab vs. placebo	Primary: DFS in PD-L1 TC ≥ 1%; Secondary: DFS, PFS, TFST
NADIM-ADJUVANT (NCT04564157)	210	Stage IB(4cm)-IIIA (AJCC 8th), *EGFR* excluded and if known *ALK/STK11/KEAP1*	Part of experimental arm	Nivolumab + carboplatin/paclitaxel vs. carboplatin/paclitaxel Q3W × 4 cycles followed by durvalumab Q4W × 6 cycles	Primary: DFS Secondary: OS, Safety
MeRmaidD-1 (NCT04385368)	322	Stage II-IIIA (AJCC 8th), EGFR/ALK excluded	Part of experimental arm	Durvalumab + platinum doublet vs. placebo + platinum doublet	Primary: DFS in MRD+ Secondary: DFS in FAS, OS in MRD+, QoL

AE, adverse event; ALK, anaplastic lymphoma kinase; DFS, disease free survival; EGFR, epidermal growth factor receptor; KEAP 1, Keilch like ECH Associated Protein 1; LCSS, lung cancer specific survival; MRD, minimal residual disease; OS, overall survival; PFS, progression free survival; QoL, quality of life; STK11, serine/threonine kinase 11; TFST, time to first subsequent therapy or death.

**Table 2:** Phase 2 trials of neoadjuvant immunotherapy for resectable Stage III NSCLC.

Clinical Trial	*n*	N with Stage 3	Patient Population (AJCC 7th Unless Stated Otherwise)	Intervention	Major Pathologic Response	Pathologic Complete Response
CheckMate 159 (NCT02259621)	21	7 (33%)	I (>2 cm)-IIIA	Nivolumab Q2W × 2 doses	45%	15%
NEOSTAR (NCT03158129)	ARM 1: 23	5 (22%)	I-IIIA (single station N2)	Nivolumab 3 mg/kg Day 1, 15, 29 × 2 cycles	22%	9%
	ARM 2: 21	4 (19%)	I-IIIA (single station N2)	Nivolumab 3 mg/kg Day 1, 15, 29/ipilimumab 1 mg/kg Day 1 × 2 cycles	38%	29%
ChiCTR-OIC-17013726(NCT04371796)	40	8 (20%)	IA (>2 cm)-IIIB (N2 only) (AJCC 8th), exclude *EGFR* mutated	Sintilimab Q3W × 2 doses	41%	16%
IFCT-1601 IONESCO (NCT03030131)	46	1 (2%)	IB (≥4 cm)-IIIA (non N2) (AJCC 8th)	Durvalumab Q2W × 3 doses	19%	7%
TOP 1501 (NCT02818920)	30	Not provided	Stage IB (≥3 cm)-IIIA (N0-N2)	Pembrolizumab Q3W × 2 and adjuvant pembrolizumab Q3W × 4 cycles	28%	8%
PRINCEPS (NCT02994576)	30	Not provided	IA (≥2 cm)-IIIA (non N2)	Atezolizumab × 1 dose	14%	0%
LCMC3 (NCT02927301)	181	85 (47%)	IB-IIIB (T3N2 or T4), *EGFR/ALK* excluded	Atezolizumab × 2 followed by adjuvant atezolizumab if pathologic response	20%	7%

AJCC, American Joint Committee on Cancer; ALK, anaplastic lymphoma kinase; EGFR, epidermal growth factor receptor; Q2W, every 2 weeks; Q3W, every 3 weeks.

**Table 3:** Phase 3 trials of neoadjuvant ICIs + chemotherapy for resectable Stage III NSCLC.

Clinical Trial	*n*	Patient Population (AJCC 7th Unless Stated Otherwise)	Neoadjuvant	Adjuvant	Endpoints
Checkmate 816 (NCT02998528)	350	IB-IIIA (AJCC 7th), *EGFR/ALK* excluded	Nivolumab + platinum doublet vs. platinum doublet Q3W × 3 cycles (nivolumab/ipilimumab arm closed)	None	Primary: EFS, pCR Secondary: OS, MRP, TTDM
AEGEAN (NCT03800134)	800	IIA-IIIB (N2) (AJCC 8th) Protocol amended to exclude *EGFR/ALK*	Durvalumab + platinum doublet vs. placebo+platinum doublet × 4 cycles	Durvalumab vs. Placebo Q4W × 1 year	Primary: EFS, pCR in EGFR/ALK wildtype Secondary: DFS, MPR, OS, outcome based on PDL1 expression, QoL
KEYNOTE 671 (NCT03425643)	786	IIA-IIIB (T3-4N2) (AJCC 8th)	Platinum doublet + placebo vs. platinum doublet + pembrolizumab Q3W × 4 cycles	Pembrolizumab Q3W × 13 cycles vs. placebo	Primary: EFS, OS Secondary: MPR, pCR, safety, QoL, perioperative complications
IMpower030 (NCT03456063)	450	II-IIIB (T3N2) (AJCC 8th), *EGFR/ALK* excluded	Platinum doublet vs. platinum doublet + atezolizumab Q3W × 4 cycles	Atezolizumab × 1 year	Primary: EFS Secodary: pCR, MPR, OS, DFS, ORR, QoL, safety
CheckMate77T (NCT04025879)	452	II >4 cm-IIIB (T3N2) (AJCC 8th), *EGFR/ALK* excluded	Nivolumab + platinum doublet vs. platinum doublet + placebo Q3W × 4 cycles	Nivolumab vs. placebo × 1 year	Primary: EFS. Secondary: OS, pCR, MPR, Safety

ALK, anaplastic lymphoma kinase; DFS, disease free survival; EFS, event free survival; EGFR, epidermal growth factor; MPR, major pathologic response; ORR, objective.

**Table 4:** Phase III trials of ICIs with cCRT in unresectable Stage III NSCLC.

Clinical Trial	*n*	Patient Population (AJCC 7th Unless Stated Otherwise)	Intervention	Endpoints
NCT04380636 (KEYLYNK 012)	870	Stage III unresectable	cCRT + pembrolizumab followed by pembrolizumab OR cCRT + pembrolizumab followed by pembrolizumab + olaparib vs. cCRT followed by durvalumab	Primary: PFS, OS Secondary: AEs, ORR, DoR, QoL
NCT03840902	350	Stage III unresectable	cCRT followed by durvalumab vs. cCRT+M7824 followed by 1-year M7824	Primary: PFS Secondary: OS, AEs, ORR, DoR, change in PFTs (DLCO, FEV1, FVC,6 min walk test, HR-CT)
NCT04092283 (ECOG-ACRIN EA5181)	660	Stage III unresectable	cCRT followed by durvalumab vs. cCRT + durvalumab followed by durvalumab	Primary: OS Secondary: PFS, best objective response, AEs, local progression
NCT04026412	1300	Stage III unresectable	ARM A: Nivolumab + cCRT followed by Nivolumab Plus Ipilimumab **OR** ARM B: Nivolumab Plus cCRT Followed by Nivolumab vs. ARM C: cCRT Followed by durvalumab	Primary: PFS, OS (Arm A vs. Arm C) Secondary: PFS, OS (Arm B vs. Arm C), ORR, CR rate, DoR, TTR, TTDM, AEs, SAEs, QoL

AEs, adverse events; cCRT, concurrent chemotherapy and radiation; CR, complete response; DLCO, diffusion capacity of lung carbo dioxide; DoR, duration of response; FEV1 forced expiratory volume in 1 min; FVC, forced vital capacity; HR-CT, high resolution computerized tomography; ORR, objective response rate; OS, overall survival; PFS, progression free survival; PFTs, pulmonary function tests; QoL, quality of life; SAEs, serious adverse events; TTDM, time to death or distant metastases; TTR, time to response.
